# Two-dimensional membrane as elastic shell with proof on the folds revealed by three-dimensional atomic mapping

**DOI:** 10.1038/ncomms9935

**Published:** 2015-11-19

**Authors:** Jiong Zhao, Qingming Deng, Thuc Hue Ly, Gang Hee Han, Gorantla Sandeep, Mark H Rümmeli

**Affiliations:** 1 Center for Integrated Nanostructure Physics, Institute for Basic Science, Sungkyunkwan University, Room 86175, Suwon 440-746, Republic of Korea; 2 Department of Energy Science, Sungkyunkwan University, Suwon 440-746, Republic of Korea; 3 IFW Dresden, Institute of Solid State Research, PO Box 270116, Dresden D-01171, Germany

## Abstract

The great application potential for two-dimensional (2D) membranes (MoS_2_, WSe_2_, graphene and so on) aroused much effort to understand their fundamental mechanical properties. The out-of-plane bending rigidity is the key factor that controls the membrane morphology under external fields. Herein we provide an easy method to reconstruct the 3D structures of the folded edges of these 2D membranes on the atomic scale, using high-resolution (S)TEM images. After quantitative comparison with continuum mechanics shell model, it is verified that the bending behaviour of the studied 2D materials can be well explained by the linear elastic shell model. And the bending rigidities can thus be derived by fitting with our experimental results. Recall almost only theoretical approaches can access the bending properties of these 2D membranes before, now a new experimental method to measure the bending rigidity of such flexible and atomic thick 2D membranes is proposed.

## Abstract (web summary)

The out-of-plane bending of 2D materials is an important factor to consider for applications, but is challenging to probe experimentally. Here, the authors map the out-of-plane bending in graphene and other 2D materials using high resolution electron microscopy.

## Graphical abstract

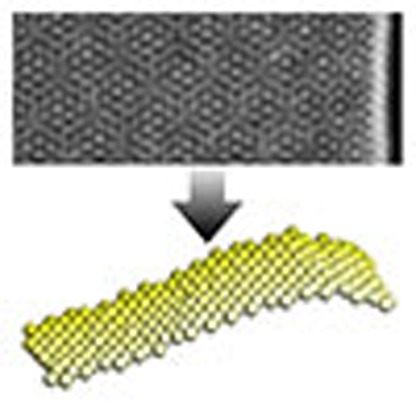

Out-of-plane bending properties are of great significance for graphene as well as two-dimensional (2D) transition metal dichalcogenide (TMD) membranes such as 2H-MoS_2_, WS_2_ and WSe_2_ (ref. 1), because it can explain the morphology of such materials under external fields and is thus important with regards to their use in applications^2^. Here we propose a simple approach to three dimensionally map the folded edges of these 2D membranes at the atomic scale, based on the high-resolution transmission electron microscopy (HRTEM). A quantitative comparison with the continuum mechanics shell model confirms that all the bending behaviour of the investigated 2D materials can be well explained by the linear elastic shell model.

Excellent elasticity for these 2D materials has been demonstrated in which they approach their theoretical in-plane tensile strength and exhibit ultrahigh in-plane modulus^3^. Nano-indentation tests on suspended membranes using atomic force microscope tips^4^ or direct in-plane tensile testing involving microelectromechanical systems gave rather scattered results for the Young's modulus and strength, which suggest many unknown factors in sample preparation, loading system and, in particular, identifying the correct thickness for these membranes^5^. In most of these measurements, a linear and isotropic elasticity were assumed. However, some theoretical studies using density functional theory (DFT), molecular dynamics or microscopic analytical derivation from the empirical interatomic potential suggest a nonlinear and anisotropic elasticity in large strain regimes^3^.

In contrast to the above in-plane tests, the bending test (for flexural rigidity and flexural strength), which is also an important elastic measurement for thin plates or shells^6^, is still lacking for most of the 2D membranes. The applicability of elastic shell theory on 2D membranes, which are only 1 to 3 atoms thick, has not been verified experimentally, although various theoretical studies have looked at the problem^7^,^8^. The primary experimental difficulty is that such 2D membranes are too flexible, and their bending rigidities are too small as compared with their in-plane rigidities. One experimental work on the nanometer scale rippling structure of graphene shows violation to the continuum mechanics shell model^9^. Another recent study reported the bending stiffness measured in monolayer graphene by the thermal method and laser method^10^. Moreover, the direct observation of the cross-sections of these 2D membranes after bending is either inaccurate by atomic force microscope^11^ or interfered by supporting layers in transmission electron microscopy (TEM)^12^, thus no intrinsic mechanical properties can be derived.

The naturally folded or buckled structures of 2D membranes have been observed in many previous reports^13^,^14^, however, their focus was mainly on the stacking order after folding. In addition, one of the studies also conducted in an investigation of the 2D strain mapping of the folded graphene using Geometric Phase Analysis^15^. A few studies have correlated the van der Waals (vdW) interaction and critical diameter for collapsed carbon nanotubes^16^,^17^. However, in this study, based on a 3D atomic mapping by using high-resolution HRTEM and scanning TEM (STEM), we confirmed the continuum mechanics model and derived the bending rigidity of 2D materials.

## Results

### TEM imaging of the 2D membranes

The 2D membranes we investigated are chemical vapour deposition (CVD) fabricated and transferred onto standard TEM grids^18^,^19^. JEOL ARM-200F TEM with CEOS probe aberration corrector was used for high-resolution HRSTEM annular dark-filed (ADF) imaging and a JEOL 2010F TEM equipped with CEOS image aberration corrector was employed for HRTEM imaging, both with an acceleration voltage of 80 kV (see Methods and Supplementary Discussion for details).

**Figure 1:**
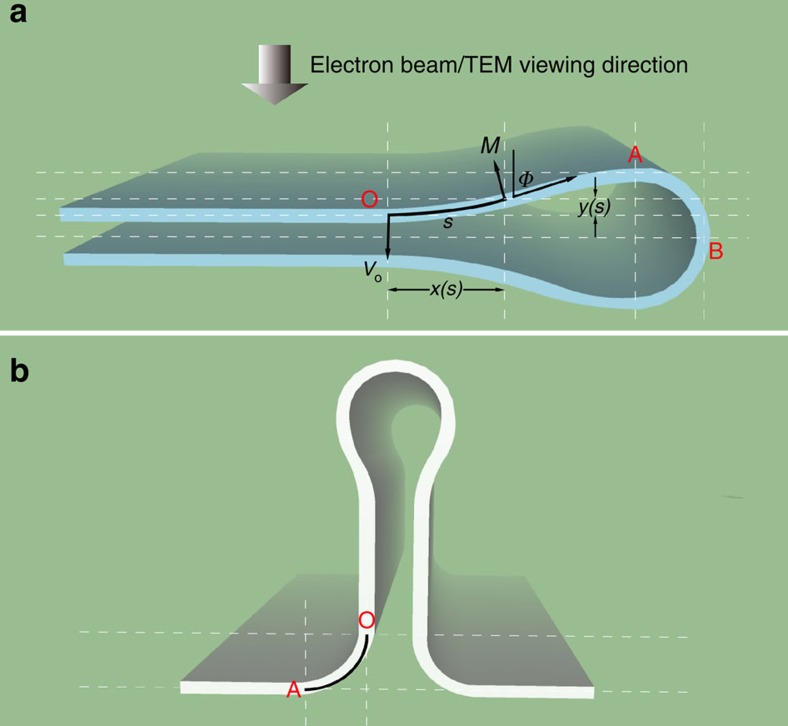
Scheme of folded structures of 2D membranes. (**a**) A horizontal aligned fold with viewing direction upside down in TEM. (**b**) A vertical buckled fold.

**Figure 2:**
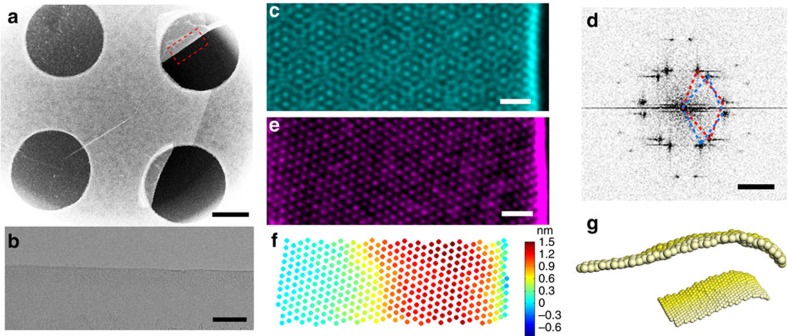
Analysis on the high-resolution image of folds and 3D reconstruction. (**a**) Low-magnification TEM image of the WSe_2_ membrane on grids, the dashed red rectangle highlights the folded part, scale bar, 500 nm. (**b**) TEM image shows the natural folded membrane without any inclusions, scale bar, 5 nm. (**c**) ADF image of one chiral WSe_2_ fold. False colour is applied, scale bar, 1 nm. (**d**) FFT of the image in **c**, which has two sets (red and blue) of spots corresponding to the up and bottom halves of the fold, scale bar, 3 nm^−1^. (**e**) Inverse FFT image for half of the WSe_2_ fold in **c**, scale bar, 1 nm. (**f**) Height (*z*) profile picture for all the W atoms in **e** after 3D mapping. (**g**) Derived 3D atomic model for half of the WSe_2_ fold, only W atoms shown here.

The schemes of the folded and buckled (vertically folded) structures of 2D membranes are presented in Fig. 1a,b, respectively. It is not hard to find regions of sample with folded areas for all of our observed 2D specimens. All the examined areas are monolayers, with some parts folded during transfer process or due to crack (Fig. 2a). Figure 2b shows one WSe_2_ fold, which has no inclusions inside, leaving the folded area an intrinsic structure. A typical high-resolution ADF image of the folded edge of WSe_2_ is presented in Fig. 2c. It shows a Moiré Pattern from one folded chiral edge (whereas zigzag and armchair folds are achiral). The fast Fourier transform (FFT) of this image shows similar streaking of the certain reflexes in a particular direction (west to east in this case; Fig. 2d). Such streaking of reflexes can also be found with carbon nanotubes. The FFT consisting of two sets of spots corresponding to the upper and lower half of the fold (see Supplementary Figs 1 and 2 for details). To view a single side of the fold, we carefully select the reflexes corresponding to the chosen side (forming a selective mask) and then apply inverse FFT. This process provides a micrograph comprised solely of one side chosen from the fold (Fig. 2e). In this reconstructed ADF image, the position of the brightest spots reflect the real atomic positions of Tungsten (W) atoms. The Se atoms (which have a reduced Z contrast) are not well resolved in part because of the relative tilt of the two Se atoms in one column in the folded region. However, to determine the strain state of the lattice, a clear knowledge of the positions of only one kind of atom (W in this case) is sufficient. The following analysis are all based on the positions of transition metal atoms in TMD membranes, whereas for graphene because the images were collected in HRTEM, the nodes of Voronoi cell in the honeycomb lattice are analysed.

### 3D reconstruction from HR(S)TEM images

Therefore, positions of all the W atoms in WSe_2_ sample (Fig. 2) can be determined by a 2D peak fitting approach (see Supplementary Figs 3–5 and Supplementary Discussion for details). However, they represent a project image onto 2D space (*x* and *y* direction). The *z* coordinate can be determined from the local curvature-dependent (considering only a single curvature direction in the fold, *κ*_x_) W–W bond length (*d*_w–w_) as (*x*′-*x*)^2^+(*y*′-*y*)^2^+(*z*′-*z*)^2^=*d*_w–w_(*κ*_x_)^2^, where (*x*, *y*, *z*) and (*x*′, *y*′, *z*′) are the coordinates for two W atoms, respectively. DFT calculations on various TMD nanotubes have already determined the equilibrium state of the W–W bond length as a function of curvature *κ*_x_ (or the diameter; see refs 20, 21 and Supplementary Figs 6–10 for details). When the diameter is larger than 2 nm, there is less than a 3% difference compared with free 2D materials. We then applied this DFT-derived *d*_w–w_(*κ*_x_) relationship into our 3D reconstruction of the folds. The reference point (Point O in Fig. 1a) relatively far away from the folded edge is selected first. Then the *z* coordinates for the W atoms are calculated from the flat area towards the folded edge. The initial W–W bond length is selected as the equilibrium bond length measured from the flat area. At least two nearest W–W bonds are considered and averaged during reconstruction. Because the obtained relative *z* coordinate is quadratic, the sign(+ or −) of (*z*′-*z*) is determined from the first derivative of (*z*′-*z*)^2^. After a single calculation cycle, the local curvature *κ*_x_ is then calculated for each W atom. In the next cycle, the DFT-derived relationship *d*_w–w_(*κ*_x_) from last cycle is applied. This iteration is then repeated until the difference between two cycles of *d*_w–w_(*κ*_x_) is less than 0.05 Å. This self-consistent calculation can yield the precise *z* coordinate, which both satisfies the *d*_w–w_(*κ*_x_) relationship and the TEM-derived 2D positions (Fig. 2f,g). The calculation was developed and performed using Matlab (see Supplementary Fig. 10 and Supplementary Discussion for details). Moreover, we have carefully investigated the error introduced by FFT filtering of the random noise in the previous separation step on two halves of the folds, which is proved to have no systematic error in our mechanical analysis (Supplementary Figs 4–6 and Supplementary Discussion).

### Comparison with continuum mechanics models

Previous studies on folded structures used the small deflection approximation, which is not appropriate for folds^22^,^23^. In the framework of linear elastic continuum mechanics, bending stiffness/rigidity (*D*) is the resistance against bending deformation, it equals to the ratio between bending moment (*M*) and curvature (*κ*). For one dimensional pure bending as in Fig. 1a, the bending moment (*M*) is related to the curvature,


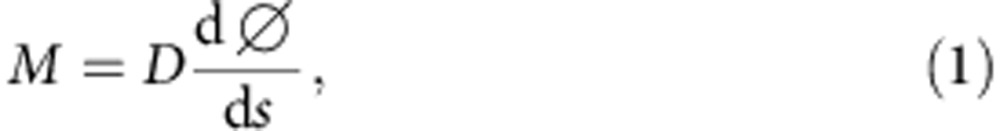


where *D* is the bending rigidity or bending stiffness, 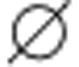 is angle of the section *s*. As the moment balance at the initial point O (Fig. 1a),


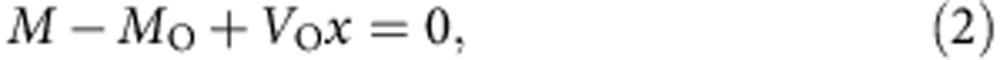


*M*_O_ is the bending moment at point O, *V*_O_ is the shear force exerted at O point, the direction is perpendicular to the 2D membrane. From the geometry in Fig. 1a, we know d*x*/d*s*=cos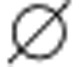, and by differentiating equation (2) and use equation (1), we can get^24^


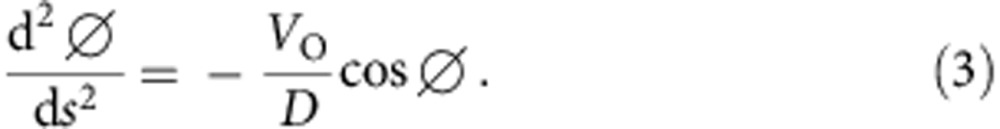


Note one key boundary condition at the initial bending point O comes from the balance of the adhesion energy between two layers and strain energy caused by bending (see Supplementary Discussion for details), which yield a simple relationship^25^


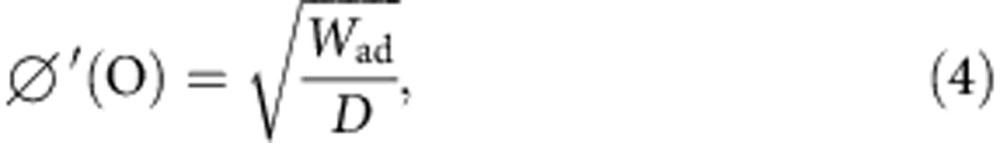


where *W*_ad_ is the adhesion energy between the folded two layers. We then use a similar approach as found in ref. 24 to normalize equation (3). After integration on equation (3) and applying the boundary condition in equation (4), we get


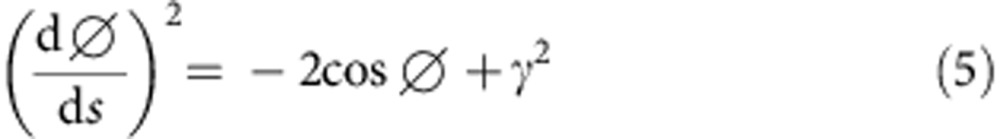


where, 
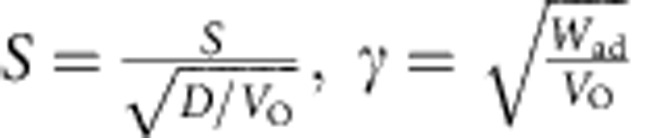
, they are dimensionless values, and *x* and *y* are also normalized as 
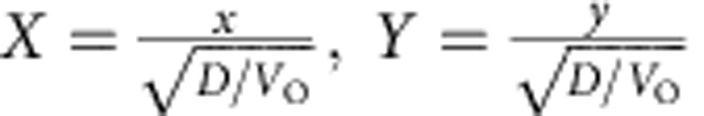
. And using boundary conditions at point O and B (Fig. 1a), 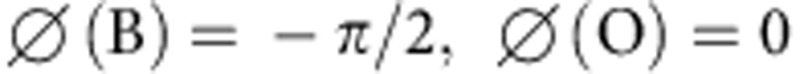, equation (5) can now be numerically solved.

**Figure 3:**
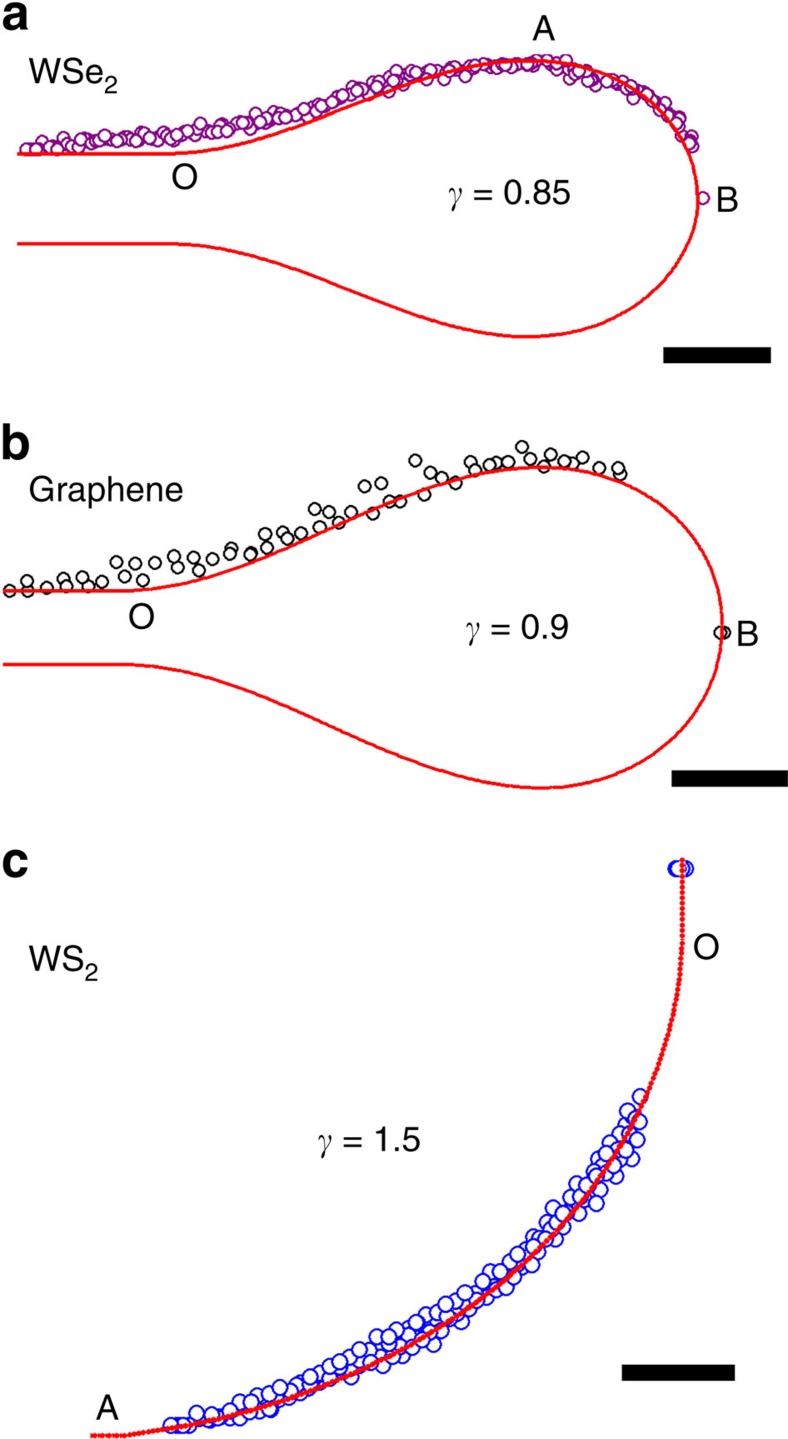
Comparison with the continuum mechanics models. (**a**) The horizontal folds of WSe_2_, scale bar, 1 nm. Open circles stand for the atomic positions, and red solid line is the elastic models calculated by continuum mechanics (horizontal cross-section view), same for the cases in **b**,**c**. (**b**) Horizontal folds of graphene, scale bar, 0.5 nm. (**c**) Vertical fold of WS_2_, scale bar, 1 nm.

Varying *γ* leads to different shapes of corresponding folds as show in Fig. 3. Our experimental reconstructed 3D folded structures for different kinds of membranes are then fitted with the continuum mechanics models. For instance, for WSe_2_ and Graphene (Fig. 3a,b), we find the cross-section contour can all be fitted very well with the linear elastic models, with different *γ*. For all of our bending modulus measurements, we have selected the samples that both sides of the folds can be fitted well with the same continuum mechanics model (Supplementary Fig. 11). In addition to the horizontal folds, the continuum mechanics model can also be applied to vertical folds (buckled) for WS_2_ (Figs 1b and 3c). However, in this case the boundary condition 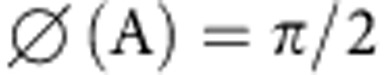 is used.

**Figure 4:**
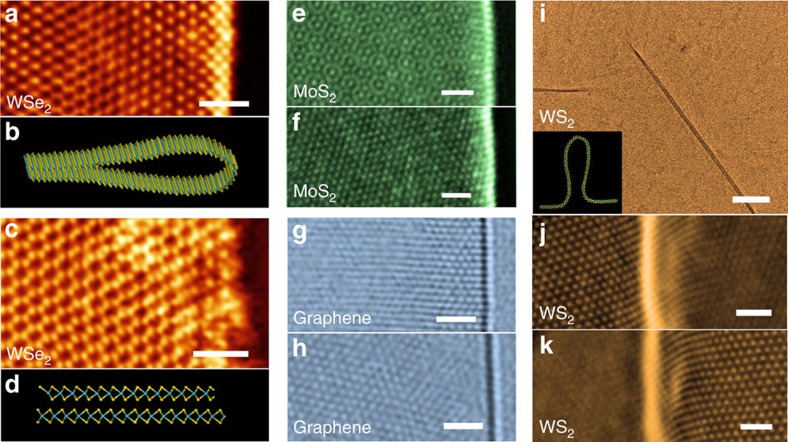
High-resolution (S) TEM images for various folded structures. (**a**) ADF image for one armchair WSe_2_ fold, scale bar, 1 nm. (**b**) Atomic model for **a**. (**c**) The cracked WSe_2_ fold (strain released), which has a nearly perfect AB stacking order between the up and bottom layers, scale bar, 1 nm. (**d**) Atomic model for **c**. (**e**,**f**) Original (**e**) and separated half ADF images (**f**) for one chiral MoS_2_ fold, similar to Fig. 2c,d, scale bars, 1 nm. (**g**,**h**) The HRTEM images for zigzag (**g**) and chiral (**h**) monolayer graphene folds, scale bars, 1 nm. (**i**) Low-magnification TEM image for WS_2_ vertical fold, model shown in inset, scale bar, 100 nm. (**j**,**k**) High-resolution ADF images for the vertical fold, the membrane has some rippling so the left (**j**) and right part (**k**) are not on the same height and cannot be focused in the same time thus are presented in two panels, scale bars, 1 nm.

We examined folds with different chiralities for WSe_2_, WS_2_, MoS_2_ and graphene, including horizontal folded structures and vertical folded structures. The ADF image for armchair fold of WSe_2_ is shown in Fig. 4a,b, whereas perfect AB stacking is obtained if the armchair fold is collapsed (Fig. 4c,d). Figure 4e shows the ADF image of one chiral MoS_2_ fold edge, and the separated half fold (Fig. 4f). For graphene, the HRTEM images for zigzag and one chiral fold are shown in Fig. 4g,h, respectively. At last, Fig. 4i–k presents the ADF image for vertical folded WS_2_. It should be noted that pure armchair or zigzag folds have only one set of reflexes in FFT and is hard to separate into two halves, for these cases very small chiral angle (<3°) samples are analysed (see Supplementary Fig. 2 and Supplementary Discussion).

By fitting all of the experimental measured structures with the linear elastic shell model, not only can we check the applicability of the elastic shell model, but also from the ratio of *x*/*X* (measured size or dimensions of the folds/non-dimensional values for size in the normalized model) and *γ*, the *W*_ad_/*D* can be obtained. Regarding the *W*_ad_, the adhesion energy for the bilayer 2D materials in the folded structures, there does exist experimental data for graphene^26^,^27^, which show similar values to theoretically computed values^28^,^29^. As for TMD materials, *W*_ad_ has still not been measured experimentally. Therefore, we employed the DFT-vdW corrected method^30^ to obtain the adhesion energy (*W*_ad_) for TMD materials, MoS_2_, WS_2_ and WSe_2_, for different chiralities (Supplementary Fig. 12). The AB stacking and chiral (rotational) stacking order for these TMD materials do not show much difference in *W*_ad_. Then with our experimentally measured values, the bending rigidity(stiffness) can be calculated. The measured *D* for different 2D membranes, and different chiralities, along with the fold type are summarized in Table 1. The errors of *x*/*X* and *D* in Table 1 are the standard deviations from at least three measurements (from three high-resolution images) on the same fold, and the final *D* values are the mean value. And on the same fold, the distance between each two different places that we measured is fairly far (>200 nm), compared with the dimension of each folds we are focusing on (<10 nm), therefore we consider the sampling areas as individual separate folds with the same chirality. By multiple times of measurements, the effect of local rippling of these 2D membranes can be reduced.

## Discussion

The measured bending rigidity for graphene is 1.8–2.7 eV, close to the theoretical value of 1.4 eV (ref. 7). The bending rigidity (*D*) for TMD materials is in the range of 10–16 eV, which is about five to six times that of graphene. This is also in agreement with a theoretical determined value of 9.6 eV (ref. 8). The *D* value (bending rigidity) does not depend strongly on chirality, in agreement with the isotropic elasticity for these 2D membranes, which have higher than three-folded symmetry. The larger bending rigidity for the TMD materials as compared with graphene is in part attributed to the larger thickness of the TMDs (that is, 3 atom layers thick when considering two S/Se layers). Intrinsically, the horizontal folds have less degree of freedom than vertical ones, and when two monolayers attached to form a bilayer in the folds, the inherent rippling can be suppressed. However, this rippling can affect the vertical folds more and make more uncertainties in the bending rigidity measurement. The relatively larger uncertainty in graphene than TMD materials possibly comes from the beam-induced local atomic defects (see Supplementary Fig. 13 for details). Because we used TEM mode (unlike the STEM mode for the TMDs) to image the graphene, TEM mode using parallel beam illumination is easier to damage the sample. It should also be noted that tilted fold sample can be used for 3D reconstructions by our method (see Supplementary Fig. 14 for details), but it may cause larger uncertainties in measurement and the following fitting process.

In our observations, the largest curvatures for the bending regions are 2 nm for graphene and 0.5 nm for TMD materials. For TMD materials, this curvature corresponds to an in-plane tensile/compressive strain about 0.08 in the two S/Se layers. This value is comparable with the value for the onset of nonlinearity of the in-plane strain of TMDs^31^. Thus, our experiments have confirmed that under this curvature range the linear elastic continuum model can still work well, both for graphene (1 atomic layer) or TMD families (3 atomic layers). This linear elastic response was first raised up by Yakobson for carbon nanotubes by *ab initio* simulations^5^. It should be noted in our experiments we only consider the bending rigidity (*D*), other parameters such as the membrane thickness, elastic modulus, Poisson ratio and so on may not have a generalized value for these atomic thin membranes, possibly depending on different loading methods.

In summary, by 3D atomic mapping of the folds of 2D membranes, we experimentally confirmed the applicability of linear elastic shell model. By the new approach, we introduced the bending rigidities of these 2D membranes are derived from the folds. The improved knowledge on the out-of-plane bending of these 2D materials will aid the use and design of these materials for various applications.

## Methods

### Sample fabrication

The high-quality single-layer TMD membranes (WSe_2_, MoS_2_) were grown on sapphire substrates using CVD in a tube furnace. A description for the CVD synthesis of WS_2_ membranes will be published elsewhere. The monolayer graphene samples were also fabricated using CVD growth over a Ni/Mo substrate. The 2D membranes are transferred from the growth substrate to standard Cu TEM grid using a Polymethyl methacrylate (PMMA) transfer route.

### (S)TEM characterization

The HRTEM for graphene was performed on a JEOL 2010F transmission electron microscope equipped with a CEOS spherical aberration corrector. The TEM was operated using an 80-kV accelerating voltage, with an energy spread of 0.3 eV. The chromatic aberration Cc was ∼1 mm, and the spherical aberration, Cs, adjusted to ∼1 μm. A defocus between 4 and 5 nm was used, with a defocus spread of 3 nm. For the TMD membranes, ADF-STEM imaging was implemented using a probe aberration-corrected JEM ARM200F, operated at 80 kV. High-angle ADF images were acquired using a 20-mrad convergence angle. The beam size was about 0.15 nm. The images acquired with a medium-angle ADF detector used a convergence angle between 50 and 180 mrad with acquisition times of 32 μs per pixel.

### DFT calculations

Spin-polarized DFT calculations are performed using a plane wave basis set with the projector augmented plane wave as implemented in the Vienna *ab-initio* simulation package.The Perdew–Burke–Ernzerhof functional and a 500-eV cutoff for the plane-wave basis set were adopted in all the computations. The effect of vdW interactions is considered by using the dispersion corrected DFT (optB88-vdW function). The vacuum length between two adjacent images in the supercell is set longer than 15 Å to avoid interaction. Geometric structures are relaxed with a convergence threshold less than 10^−5^ eV in energy and 10^−3^ eV Å^−1^ in force, that is, the values of our optimized lattice parameters for bulk MoS_2_ are: *a*=3.183 Å and *c*=12.372 Å, which are in good agreement with experimental results.

### 3D reconstruction from 2D atomic images

We employed a DFT-based simulation combined technique together with HR(S)TEM imaging to derive the Z height information of the atoms (transition metal atoms in the TMD layers or nodes of Voronoi cells of graphene). The relative Z height difference between each couple of M–M atoms can be derived by simple geometry (*x*′-*x*)^2^+(*y*′-*y*)^2^+(*z*′-*z*)^2^=*d*_w–w_(*κ*_x_)^2^. The relative Z height difference can be calculated once the bond length is known, however, the bond length is dependent on the local curvature(known from DFT calculation), which is determined by the full set of *x*–*y*–*z* coordinates of the atoms after one cycle of reconstruction. Therefore, the bond length calculated by DFT is substituted into the 3D reconstruction process and iterative algorithm is applied until the final structure is consistent with the DFT calculation results.

### Fitting with the continuum mechanics models

For the horizontal folds, the atoms close to point B cannot be resolved by HR(S)TEM images because of the overlapping of atoms there, and there is one gap close to the edge. However, by 3D reconstruction and TEM images, we can know exactly the highest point A and the relative position from O (flat area), and also know the *x*,*y* coordinates of the atoms at edges (point B; Fig. 1a). We check the two following values from experiments to the continuum mechanics models with different *γ*, Δ*y* (OA)/Δ*x* (AB) and Δ*y* (OA)/Δ*y* (AB), the goodness of fitting is evaluated by the simultaneously fitting of both values. We avoid to use Δ*x* (OA) in our fitting because it has a larger uncertainty. Thereby, the experimental 3D structure is put together with the continuum mechanics model to check the overall coincidence with each other. The same procedure is applied to the vertical fold and the overall section OA is fitted with the continuum mechanics model.

## Additional information

**How to cite this article:** Zhao, J. *et al.* Two-dimensional membrane as elastic shell with proof on the folds revealed by three-dimensional atomic mapping. *Nat. Commun.* 6:8935 doi: 10.1038/ncomms9935 (2015).

## Supplementary Material

Supplementary Information Supplementary Figures 1-14, Supplementary Discussion and Supplementary References

## Figures and Tables

**Table 1:** Experimental and theoretical derived parameters for 2D membranes.

**Material**	**Fold type**	**Chirality (chiral angle in brackets)**	***W***_**ad**_ **(calculated/measured (eV Å**^**−2**^**))**	***x*****/*****X*** **(measured (Å))**	***γ*** **(fitted)**	***D*** **(bending rigidity (eV))**
WSe_2_	Horizontal	Chiral (8°)	0.022	19.8±1.5	0.85	11.9±1.8
WSe_2_	Horizontal	Zigzag (2.5°)	0.022	19.6±1.9	0.82	12.4±2.2
WSe_2_	Horizontal	Armchair (3°)	0.022	19.2±2.0	0.84	11.5±2.4
WS_2_	Horizontal	Chiral (10°)	0.021	25.0±3.8	0.88	16.9±4.8
WS_2_	Horizontal	Chiral (25°)	0.021	20.7±1.1	0.85	12.4±1.2
WS_2_	Vertical	Chiral (15°)	0.021	39.7±8.8	1.5	11.0±4.9
MoS_2_	Horizontal	Chiral (15°)	0.022	18.3±0.6	0.85	10.2±0.6
Graphene	Horizontal	Zigzag (3°)	0.014 (17)	12.9±1.4	0.92	2.75±0.60
Graphene	Horizontal	Chiral (15°)	0.014 (17)	10.4±0.5	0.9	1.87±0.15

2D, two-dimensional.
