# Supplementary material for: Two-dimensional membrane as elastic shell with proof on the folds revealed by three-dimensional atomic mapping
Source: Nat Commun. 2015 Nov 19;6:8935. doi: 10.1038/ncomms9935 (PMC4673832; doi:10.1038/ncomms9935)
Supplement: Supplementary Information — Supplementary Figures 1-14, Supplementary Discussion and Supplementary References [file ncomms9935-s1.pdf]

Supplementary Figures

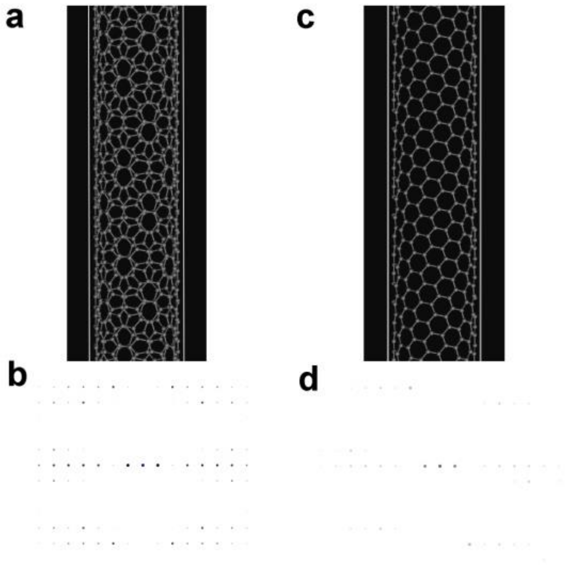

Supplementary Figure 1 Structures and simulated diffraction patterns for a (10,5) carbon nanotube and half nanotube.

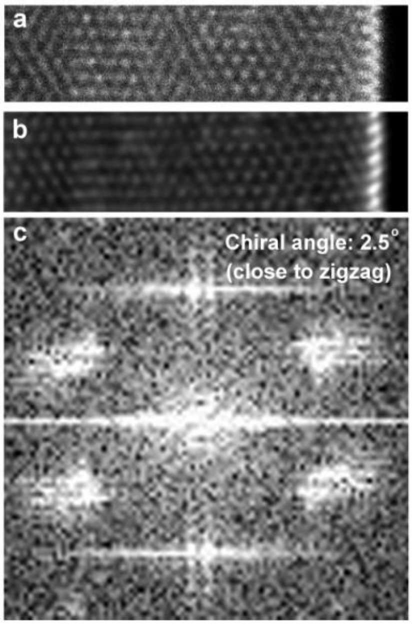

Supplementary Figure 2 a, 2.5°chiral angle WSe_2 fold image. **b**, separated half fold image. **c**, FFT.

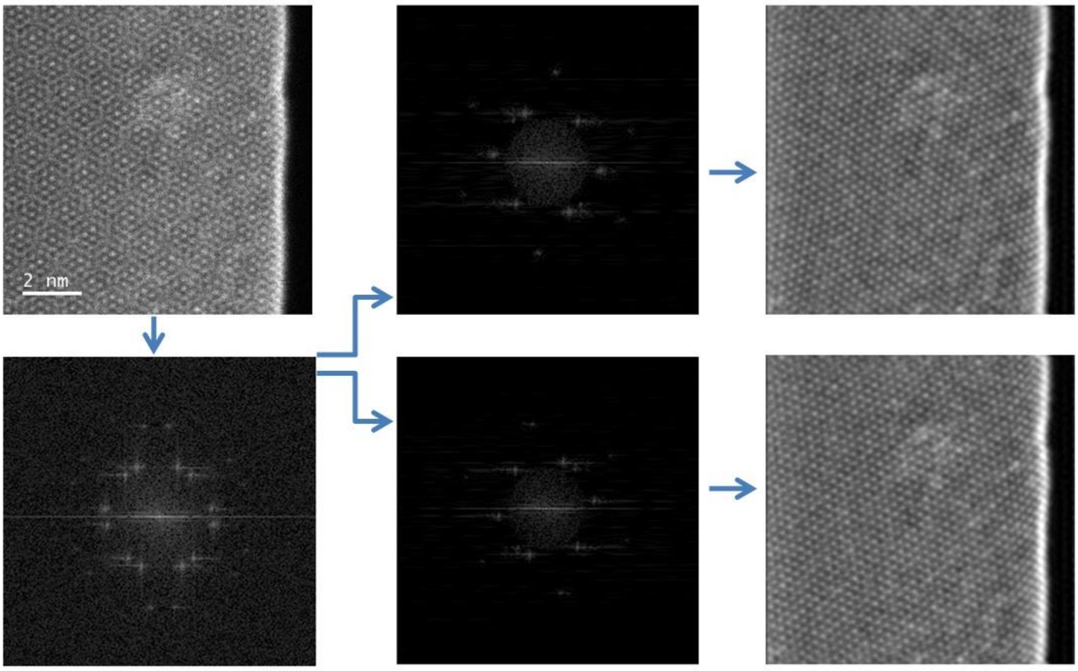

Supplementary Figure 3 Procedures to separate the upper and lower part of the folds. 1st step, FFT; 2nd step, mask and selection of corresponding reflexes; 3rd step, inverse FFT.

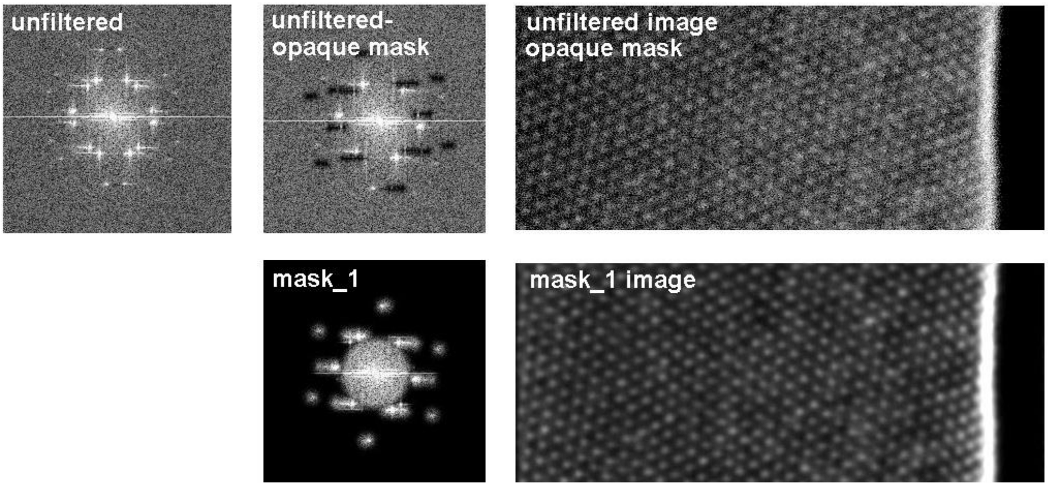

Supplementary Figure 4 Comparison of the different filtering methods.

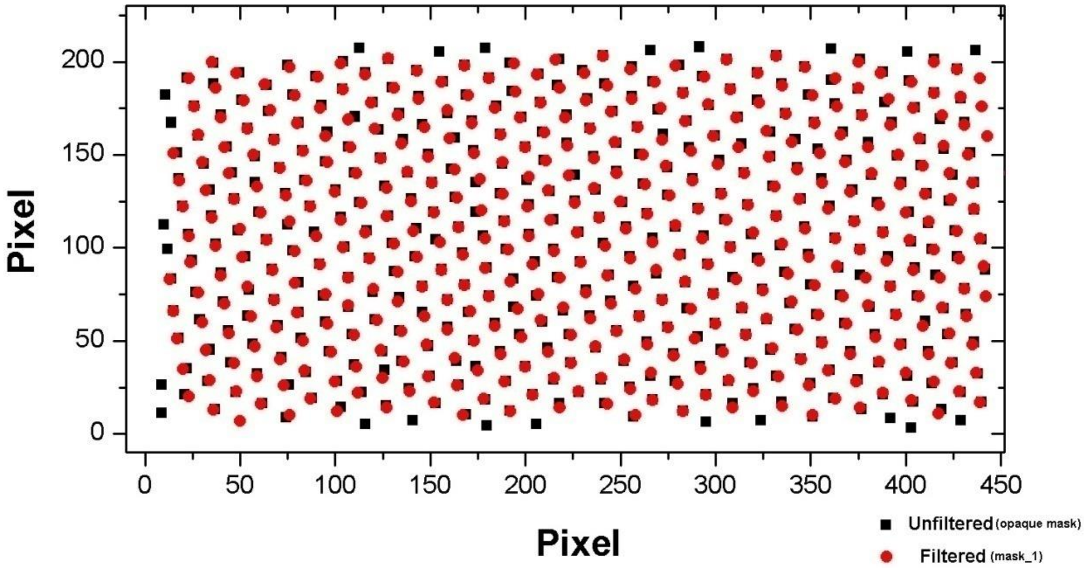

Supplementary Figure 5 Atomic positions determined by different filtering methods.

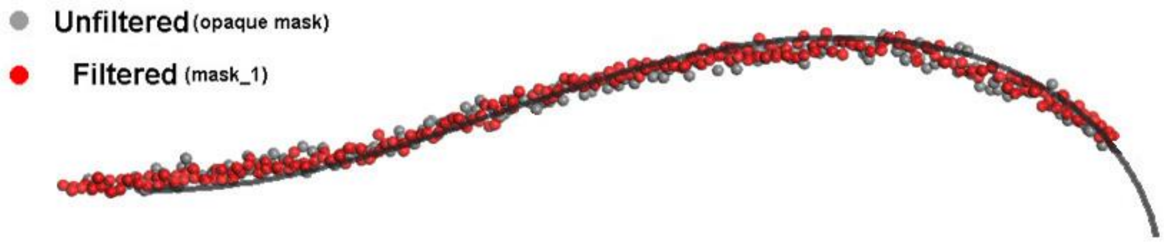

Supplementary Figure 6 Reconstructed folded structure using different filtering methods.

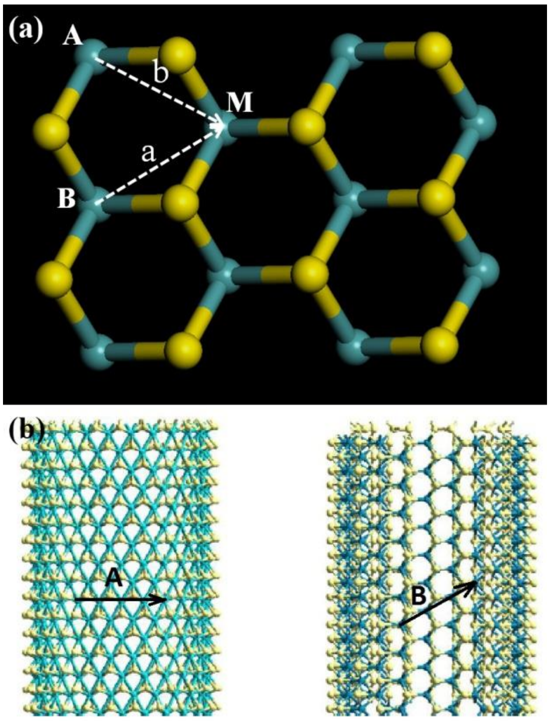

Supplementary Figure 7 a, The atomic models of TMD materials (green: transition metal atoms Mo or W, yellow: Se or S) **b**, nanotubes.

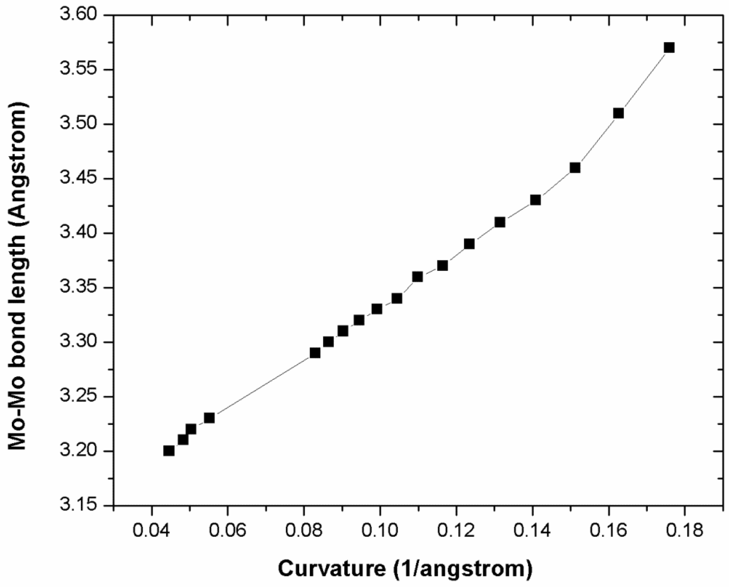

Supplementary Figure 8 The DFT calculated M-M bond length as a function of the local curvature.

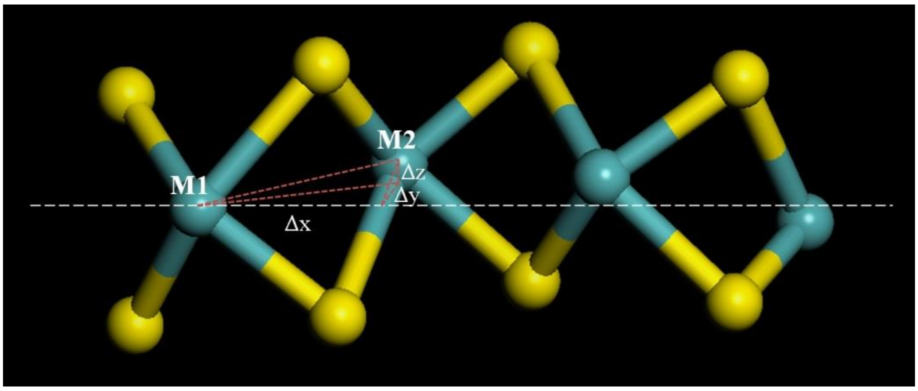

Supplementary Figure 9 Scheme showing the geometry relationship between the bond length $d_{M1\text{-}M2}$ and the relative coordinates (Δx, Δy, Δz).

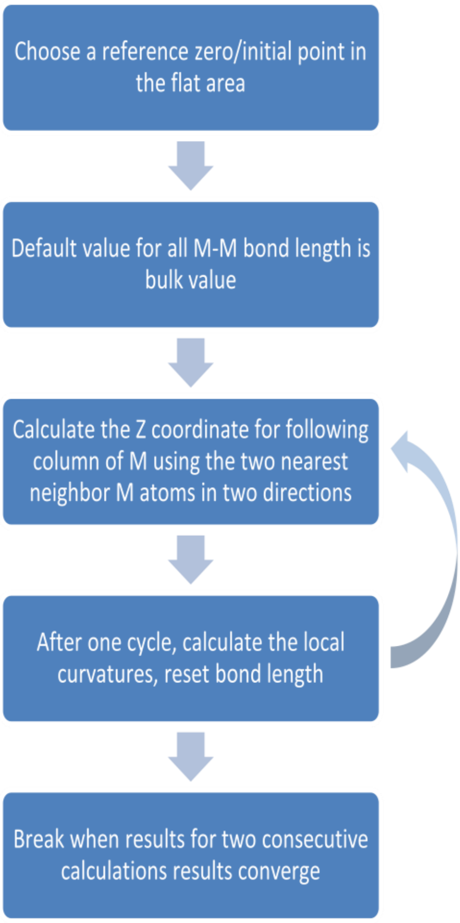

Supplementary Figure 10 The iterative algorithm for the calculation of Z coordinates of the atoms and 3D reconstructions.

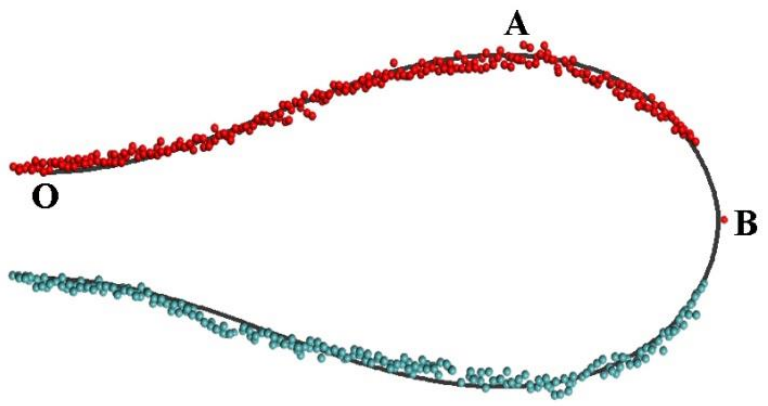

Supplementary Figure 11 3D reconstruction of both sides(upper and lower) of the fold and fitting with the same continuum mechanics model.

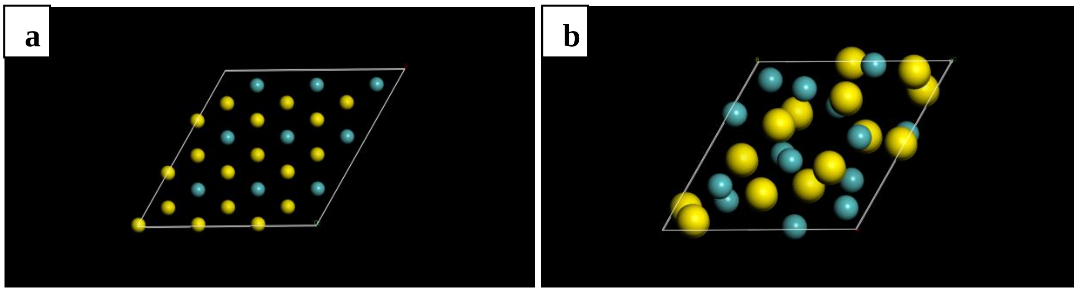

Supplementary Figure 12 **a**, The optimized unit cell structure for AB stacking MoS_2 **b**, structure for 21° chiral stacking MoS_2, S atoms (yellow) and Mo atoms (green).

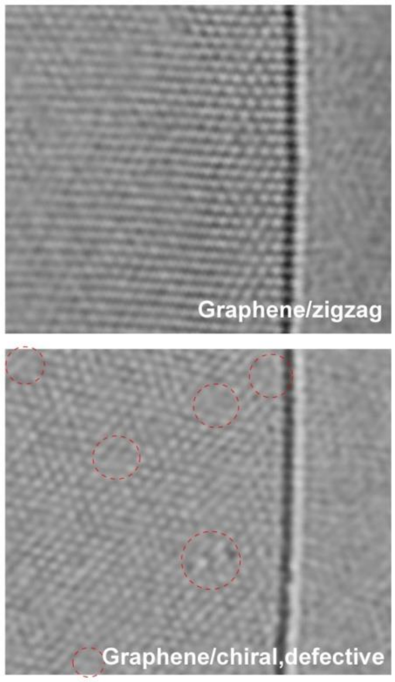

Supplementary Figure 13 The TEM images of graphene folds without(upper) or with(lower) atomic defects.

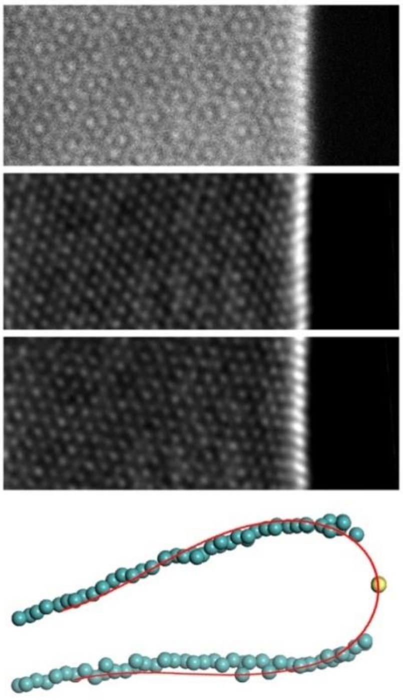

Supplementary Figure 14 The original, separated STEM images and 3D reconstruction of one tilted MoS_2 fold.

Supplementary Discussion

Synthesis of 2D Materials and TEM sample preparations

The high quality single layer transition metal dichalcogenide (TMD) membranes (WSe_2, MoS_2) were grown on sapphire substrates using chemical vapor deposition (CVD) in a tube furnace. Details of the synthesis procedure can be found elsewhere[1,2]. A description for the CVD synthesis of WS_2 membranes will be published elsewhere. The monolayer graphene samples were also fabricated using CVD growth over a Ni/Mo substrate[3]. The 2D membranes are transferred from the growth substrate to standard Cu TEM grid using a PMMA transfer route as described[2,3].

Specifications of TEMs

The HRTEM for graphene was performed on a JEOL 2010F transmission electron microscope equipped with a CEOS spherical aberration corrector[4]. The TEM was operated using an 80 kV accelerating voltage, with an energy spread of 0.3eV. The chromatic aberration Cc was around 1mm, and the spherical aberration, Cs, adjusted to ~1μm. A defocus between 4~5nm was used, with a defocus spread of 3nm.

For the TMD membranes, annular dark field (ADF)-STEM imaging was implemented using a probe aberration corrected JEM ARM200F, operated at 80 kV. High angle annular dark field (HAADF) images were acquired using a 20 mrad convergence angle. The beam size was about 0.15nm. The images acquired with a medium-angle annular dark field detector used a convergence angle between 50 mrad to 180 mrad with acquisition times of 32 μs per pixel.

Procedures to obtain the upper/lower part of the folds and error analysis

By continuum elasticity theory, a fold structure has pure bending strain without any shear strain, which is similar to the tube like structure. The simulation of diffraction of tube structure is shown in Supplementary Figure 1b,d and the simulated diffraction pattern for the structures in Supplementary Figure 1a,c, respectively.

For a chiral tube, the Fourier components will split into two sets which correspond to the upper and lower half of the tube. The folded structure can be divided into several arc sections of tube-like-structures just with different diameters(curvatures), but the main feature is the same, that both contain several equatorial streaks parallel to the folding direction. However, if the fold is purely zigzag or armchair, this separation cannot work. Therefore, in our analysis, we used the fold structures very close to (2.5~3° chiral angle) zigzag or armchair to measure the bending properties of these two directions (see supplementary Figure 2). Supplementary Figure 2a, b and c are the original image, separated half fold image and the FFT, respectively.

In our experiments, along these zigzag or armchair folds, we usually can find some places with very slight chiral angles which makes it possible to separate the two halves and then do 3D reconstructions. The actual chiral angles are shown besides final bending modulus results (Table 1 in main text) for the zigzag and armchair fold cases. The Supplementary Figure 3 presents the separation of one chiral fold image of WSe_2.

The purpose for us to do the FFT filtering here is (1) to filter the high frequency noise in the STEM images, (2) to reduce some possible noisy background contrast induced by some amorphous absorption on the sample surface during scanning inside the TEM column, thus to increase the peak finding accuracy in the following steps. Therefore, the images are Fourier filtered, using previously reported methods[5]. In the following we carefully investigate this filtering effects. The masking areas are mainly related to the Fourier components (elongated streaks due to the continuous strain effect on this fold) of these monolayer 2D materials, see Supplementary Figure 4 for one WSe_2 fold case.

In the first case, we just used an opaque mask (unfiltered-opaque mask) only subtracting the Fourier components from the other half of fold but include all the high frequency and low frequency noises. In the inverse FFT image we can see much higher noise level than the filtered one. In the second case, we apply mask(mask_1) on the Fourier components related to

the under investigation half fold and remove the high frequency part. The unfiltered image with opaque mask and mask_1 images are both applied with a peak finding procedure based on maximum searching, peak area merging and cropping, and 2D Gaussian fitting using Matlab script. For the unfiltered case (first case), some of the peaks cannot be fitted by the good Gaussian shape, and we just used the local maximum as the atom position in 2D. The identified 2D atomic positions for these two images are presented in Supplementary Figure 5.

We can see that for the filtered and unfiltered cases most of the atomic positions are quite close, especially for the positions near the edge position very good correspondence is still achieved, and we didn't see obvious effects on filtering, but only can see a few atomic positions are a bit deviated.

Then we use our algorithm to do the 3D reconstruction using these two above sets of atomic positions(Supplementary Figure 6). From this cross section view of the fold for these two cases, the determined atomic positions for the unfiltered case is more scattered than the filtered case due to the randomness caused by noise. However, the main shape of the fold can be both well fitted with the continuous mechanics model (γ=0.85). And for the real case, the more smoothly changing structure(filtered case) should be closer to the real situation, and that's the main reason we use filtering before doing analysis. On the other hand, because our modelling depends on the relative position between the atomic positions, and the total dimension of these fold areas(total number of atoms also fixed) is fixed, the randomness in peak finding caused by the noise or amorphous will be naturally compensated in the reconstruction (for details can see Appendix at the end of this section), this is also one advantage of our method. And we checked the filtering effect for the other samples of WSe_2, MoS_2, WS_2 and graphene, all of them are fitted well by the similar continuum mechanics model and remain stable in the final fitting parameters.

The error caused by noise on 3D reconstruction

Originally, $Z_i=Z_{i-1}+(d^2-(X_{i-1}-X_i)^2-(Y_{i-1}-Y_i)^2)^{1/2}$,

$Z_{i+1}=Z_i+(d^2-(X_i-X_{i+1})^2-(Y_i-Y_{i+1})^2)^{1/2}$,

so original $(Z_{i+1}-Z_{i-1})=(d^2-(X_i-X_{i+1})^2-(Y_i-Y_{i+1})^2)^{1/2}+(d^2-(X_{i-1}-X_i)^2-(Y_{i-1}-Y_i)^2)^{1/2}$.

If atom position(X_i) is deviated by δ (randomness) from original position,

Then, $Z_i=Z_{i-1}+(d^2-(X_{i-1}-(X_i+\delta))^2-(Y_{i-1}-Y_i)^2)^{1/2}$,

$Z_{i+1}=Z_i+(d^2-((X_i+\delta)-X_{i+1})^2-(Y_i-Y_{i+1})^2)^{1/2}$,

and $Z_{i+1}-Z_{i-1}=(d^2-(X_{i-1}{}^2+(X_i+\delta)^2-2X_{i-1}(X_i+\delta))-(Y_{i-1}-Y_i)^2)^{1/2}+(d_2-(X_{i+1}{}^2+(X_i+\delta)^2-2X_{i+1}(X_i+\delta))-(Y_{i-1}-Y_i)^2)^{1/2}$,

terms contain higher order of δ can be omitted, and assume the strain distribution is continuous and smooth, where $X_i-X_{i-1}=X_{i+1}-X_i+\Delta x$ and $Y_i-Y_{i-1}=Y_{i+1}-Y_i+\Delta y$, here Δx and Δy are small values compared to X_i-X_{i-1} or Y_i-Y_{i-1},

so $Z_{i+1}-Z_{i-1}= (d^2-(X_i-X_{i-1})^2-(Y_i-Y_{i-1})^2-2\delta(X_i-X_{i-1}))^{1/2}+(d^2-(X_{i+1}-X_i)^2-(Y_{i+1}-Y_i)^2+2\delta(X_{i+1}-X_i))^{1/2}$

$=(d^2-(X_i-X_{i-1})^2-(Y_i-Y_{i-1})^2)^{1/2}-\delta(X_i-X_{i-1})/(d^2-(X_i-X_{i+1})^2-(Y_i-Y_{i+1})^2)^{1/2}+(d_2-(X_{i-1}-X_i)2-(Y_{i-1}-Y_i)^2)^{1/2}+\delta(X_{i+1}-X_i)/(d^2-(X_{i+1}-X_i)^2-(Y_{i+1}-Y_i)^2)^{1/2}$

$=$ original$(Z_{i+1}-Z_{i-1})+\delta(X_{i+1}+X_{i-1}-2X_i)/(d^2-(X_{i+1}-X_i)^2-(Y_{i+1}-Y_i)^2)^{1/2}$

$Z_{i+1}-Z_{i-1}=$ original$(Z_{i+1}-Z_{i-1})-\delta\Delta_x/(d^2-(X_{i+1}-X_i)^2-(Y_{i+1}-Y_i)^2)^{1/2}$.

because Δ_x is small value compared to $(d^2-(X_{i+1}-X_i)^2-(Y_{i+1}-Y_i)^2)^{1/2}$, it's concluded that total error on the total dimension in the Z axis after reconstruction caused by noise(δ) is small which has a higher order than δ. There is a self-compensation mechanism for the random errors induced by noise in the image in this 3D reconstruction.

3D reconstruction from 2D atomic TEM images

The TEM images are 2D-projection-views (x,y directions) and a few methods can be used to reconstruct the 3D structures. The x and y coordinates of each atom can be extracted directly

from HR-ADF images or HR-TEM images. Our technique here is different from the classical tomographic multi-image approach by tilting the sample[6]. The high resolution STEM technique combined with some novel projection alignment approach can reconstruct the 3D structure at the atomic scale for parts of a nanoparticle[7] or dislocations[8]. Recently, the coherent TEM imaging can also reproduce the 3D atomic structure by quantitative comparisons between experimental image and simulation images[9]. However, we employed a DFT based simulation combined technique together with high resolution STEM imaging to derive the Z height information of the atoms (transition metal atoms in the TMD layers or nodes of Voronoi cells of graphene). Different chirality folded membranes (bent in one dimension) can resemble the structure of the corresponding TMD nanotubes (strain distribution are similar). DFT calculations can give the relaxed atomic structure dependent on different chirality and curvatures (or diameter of the tubes)[10]. The M-M bonding perpendicular to the folding direction is elongated as compared to the bulk value, especially in the large curved region. The zigzag nanotube has a larger effect of bond elongation than the armchair nanotube because the local curvature of the M-M bonding (curvature along the crystal direction, **a** or **b**, shown in Supplementary Figure 7a) for direction **A** is larger than for direction **B** in the same diameter of nanotubes (Supplementary Figure 7b). However, the local curvature bond length relationship can be unified (fitted by the same curve) in different chirality nanotubes thus we can directly use it for the 3D reconstructions.

Therefore, as shown by DFT calculations[10], the closest M-M bond length can be reckoned as depending on the local curvature in the bond direction. This dependence for MoS_2 is shown in Supplementary Figure 8.

With the above relationship, the relative Z height difference between each couple of M-M atoms can be derived by simple geometry (Supplementary Figure 9) using equation in the main text. The relative Z height difference can be calculated once the bond length is known, however the bond length is dependent on the local curvature which should be determined by the full set of x-y-z coordinates of the atom under consideration and the two neighbouring

atoms in that direction. The full iterative algorithm for this 3D reconstruction can be summarized as Supplementary Figure 10.

Fitting method of the experimental reconstructed 3D model and the continuum mechanics model for the folds

The 3D reconstructed model from TEM experiments is first rotated to the cross section view of the folds. For the horizontal folds (Fig. 3a, b), the atoms close to point B cannot be resolved by HR(S)TEM images because of the overlapping of atoms there, and there is one gap close to the edge. However, by 3D reconstruction and TEM images, we can know exactly the highest point A (see Fig. 1a) and the relative position from O (flat area), and also know the x,y coordinates of the atoms at edges (Point B). In all, we check the two following values from experiments to the continuum mechanics models with different γ, Δy(OA)/Δx(AB) and Δy(OA)/Δy(AB) , the goodness of fitting is evaluated by the simultaneously fit for both values, we avoid to use Δx(OA) in our fitting because it has larger error. Thereby, the experimental 3D structure is put together with the continuum mechanics model to check the overall coincidence with each other. Finally the bending rigidity values can be obtained by comparison of the experimental value and the parameter of the continuum mechanics model.

The same procedure is applied to the vertical fold (buckling) and the overall section OA (Fig. 3c) is fitted with the continuum mechanics model. Δx(OA) is the key value when calculating the bending rigidity. Supplementary Figure 11 presents one WSe_2 fold which both sides of the fold are separated and reconstructed in 3D and then fitted with one continuum mechanics model(γ=0.85), the red atoms and green atoms are reconstructed from the separated images of the same fold.

For all of our bending modulus measurements, we have selected the samples which both sides of the folds can be fitted well with the same continuum mechanics model.

DFT calculations for the adhesive energy between layers of 2D membranes

Spin-polarized density functional theory (DFT) calculations are performed using a plane wave basis set with the projector augmented plane wave (PAW) as implemented in the Vienna ab initio simulation package (VASP)[11].The Perdew-Burke-Ernzerhof (PBE) functional[12] and a 500 eV cutoff for the plane-wave basis set were adopted in all the computations. The effect of vdW interactions is considered by using the dispersion corrected DFT (optB88-vdW function)[13,14]. The vacuum length between two adjacent images in the supercell is set longer than 15Å to avoid interaction. Geometric structures are relaxed with a convergence threshold less than 10^{-5} eV in energy and 10^{-3} eV/Å in force, i.e. the values of our optimized lattice parameters for bulk MoS_2 are: a = 3.183 Å and c = 12.372 Å which are in good agreement with experimental results. The optimized AB stacking MoS_2 and √7×√7 rotational structure(chiral angle ~21°) for MoS_2 are shown in the supplementary Figure 12.

Discussions on defective and tilted 2D membranes

The lower case in Supplementary Figure 13 has more atomic defects which are highlighted by the red dashed circles. And the 3D reconstruction for the lower case cannot yield a good 3D atomic structure due to the discontinuity in the atomic rows (our method requires continuity in the neighbours of every atom) and can lead to a much lower bending rigidity with a large error. Therefore in graphene, because of the stronger beam irradiation effect which can create more defects, we can see the range in the experimental bending rigidity data (1.8~2.8eV) is relatively larger than the TMD materials.

There could be local tilt or strain in some samples, we can determine the local tilt by measuring the FFT or lattice plane distances in the high resolution images of the flat areas and thus determine the tilt angle of the sample, and then the 3D reconstruction can still be executed, like the example of MoS_2, similar to the vertical fold, 3D reconstruction of the tilted area (Supplementary Figure 14) is the same as the reconstruction in the horizontal folds, however, when determining the z height of O point (yellow atoms in above figure) during fitting, the tilted sample faces more difficulty and will lead to larger uncertainty due to

lacking of symmetry. Therefore we suggest to use the exact horizontal or vertical folded areas to measure the bending rigidity.

Additional explanations on the continuum mechanics models

Because of the folding geometry, there is shear strain whose direction is vertical at point O. The origin of shear force(V_O) is to balance the bending moment in the folded part. For this continuous mechanics modelling, there is one assumption here that adhesion energy exists on the left side of point O and becomes zero for the right side of O (Fig.1a). Near the point O, the energy caused by the detach of the two layers should be just the same as the increase in the bending energy. In other words, if the two layers of the fold detach more toward the left side of point O(Fig.1a), the adhesion energy consumed is larger than the bending energy while if the layers attach more toward the right side of O (Fig.1a) the adhesion energy accumulated is insufficient to compensate the bending strain energy.

Supplementary References

1. Huang, J.-K., Pu, J., Hsu, C.-H., Chiu, M.-H. *et al.* Large-area synthesis of highly crystalline WSe_2 monolayers and device applications. *ACS Nano* **8**, 923-930 (2014).

2. Shi, Y., Huang, J.-K., Jin, L., Hsu, Y.-T. *et al.* Selective decoration of Au nanoparticles on monolayer MoS_2 single crystals. *Sci. Rep.* **3**, 1839 (2013).

3. Rümmeli, M. H., Zeng, M., Melkhanova, S., Gorantla, S.*et al.* Insights into the early growth of homogeneous single-layer graphene over Ni–Mo binary substrates. *Chem. Mater.* **25**, 3880-3887 (2013).

4. Börrnert, F., Bachmatiuk, A., Gorantla, S., Wolf, D., *et al.* Retro-fitting an older (S) TEM with two Cs aberration correctors for 80 kV and 60 kV operation. *J. Microscopy* **249**, 87-92 (2013).

5. Jia, C., Mi, S., Urban, K., Vrejoiu, I. *et al.* Atomic-scale study of electric dipoles near charged and uncharged domain walls in ferroelectric films. *Nat. Mater.* **7**, 57-61 (2008)

6. Van Aert, S., Batenburg, K. J., Rossell, M. D., Erni, R. & Van Tendeloo, G. Three-dimensional atomic imaging of crystalline nanoparticles. *Nature* **470**, 374-377(2011).

7. Scott, M. C., Chen, C. C., Mecklenburg, Zhu, M. C. *et al.* Electron tomography at 2.4-angstrom resolution. *Nature*, **483**, 444-447(2012).

8. Chen, C. C., Zhu, C., White, E. R., Chiu, C. Y. *et al.* Three-dimensional imaging of dislocations in a nanoparticle at atomic resolution. *Nature,* **496**, 74-77(2013).

9. Jia, C. L., Mi, S. B., Barthel, J., Wang, D. W. *et al.* Determination of the 3D shape of a nanoscale crystal with atomic resolution from a single image. *Nat. Mater.* **13**, 1044-1049 (2014).

10. Zibouche, N., Kuc, A. & Heine, T. From layers to nanotubes: Transition metal disulfides TMS2. *The European Physical Journal B* **85**, 1-7 (2012).

11. Kresse, G., & Furthmüller, J. Efficient iterative schemes for ab initio total-energy calculations using a plane-wave basis set. *Phys. Rev. B* **54** 11169-11186 (1996).

12. Perdew, J. P., Burke, K. & Ernzerhof, M. Generalized gradient approximation made simple. *Phys. Rev. Lett.* **77**, 3865-3868 (1996).

13. Klimes, J., Bowler, D. R. & Michaelides, A. Chemical accuracy for the van der Waals density functional. *J. Phys.: Condens. Matter,* **22**, 022201 (2010).

14. Klimes, J., Bowler, D. R. & Michaelides, A. Van der Waals density functionals applied to solids. *Phys. Rev. B* **83**, 195131 (2011).
